# ENT-A010, a Novel Steroid Derivative, Displays Neuroprotective Functions and Modulates Microglial Responses

**DOI:** 10.3390/biom12030424

**Published:** 2022-03-09

**Authors:** Canelif Yilmaz, Thanasis Rogdakis, Alessia Latorrata, Evangelia Thanou, Eleftheria Karadima, Eleni Papadimitriou, Eleni Siapi, Ka Wan Li, Theodora Katsila, Theodora Calogeropoulou, Ioannis Charalampopoulos, Vasileia Ismini Alexaki

**Affiliations:** 1 Institute of Clinical Chemistry and Laboratory Medicine, University Clinic Carl Gustav Carus, Technische Universität Dresden, 01307 Dresden, Germany; canelif.yilmaz@uniklinikum-dresden.de (C.Y.); eleftheria.karadima@uniklinikum-dresden.de (E.K.); 2 Pharmacology Department, Faculty of Medicine, University of Crete, 71003 Heraklion, Greece; trogdak@gmail.com (T.R.); elenipapadimitriou95@yahoo.gr (E.P.); charalampn@uoc.gr (I.C.); 3 Institute of Molecular Biology and Biotechnology, Foundation for Research and Technology Hellas, 71003 Heraklion, Greece; 4 Institute of Chemical Biology, National Hellenic Research Foundation, 11635 Athens, Greece; alatorrata@eie.gr (A.L.); esiapi@eie.gr (E.S.); thkatsila@eie.gr (T.K.); tcalog@eie.gr (T.C.); 5 Center for Neurogenomics and Cognitive Research, Department of Molecular and Cellular Neurobiology, Amsterdam Neuroscience, Vrije Universiteit Amsterdam, 1081 HV Amsterdam, The Netherlands; e.thanou@vu.nl (E.T.); k.w.li@vu.nl (K.W.L.)

**Keywords:** ENT-A010, DHEA, neuroprotection, microglia, phagocytosis, hippocampus, Aβ, ΤRKA

## Abstract

Tackling neurodegeneration and neuroinflammation is particularly challenging due to the complexity of central nervous system (CNS) disorders, as well as the limited drug accessibility to the brain. The activation of tropomyosin-related kinase A (TRKA) receptor signaling by the nerve growth factor (NGF) or the neurosteroid dehydroepiandrosterone (DHEA) may combat neurodegeneration and regulate microglial function. In the present study, we synthesized a C-17-spiro-cyclopropyl DHEA derivative (ENT-A010), which was capable of activating TRKA. ENT-A010 protected PC12 cells against serum starvation-induced cell death, dorsal root ganglia (DRG) neurons against NGF deprivation-induced apoptosis and hippocampal neurons against Aβ-induced apoptosis. In addition, ENT-A010 pretreatment partially restored homeostatic features of microglia in the hippocampus of lipopolysaccharide (LPS)-treated mice, enhanced Aβ phagocytosis, and increased *Ngf* expression in microglia in vitro. In conclusion, the small molecule ENT-A010 elicited neuroprotective effects and modulated microglial function, thereby emerging as an interesting compound, which merits further study in the treatment of CNS disorders.

## 1. Introduction

Neurodegeneration and neuroinflammation are fundamental hallmarks of central nervous system (CNS) disorders [1]. Neurodegeneration refers to the impaired function and loss of neurons, which is often permanent due to the limited regenerative capacity of the adult neural system [2]. Neuroinflammation involves the inflammatory activation of glial cells, that is, microglia and astrocytes, and can have protective or destructive consequences for the neural system [1,3]. Microglia play a key role in the maintenance of homeostasis due to synaptic pruning and their clearing and neurotrophic function [3,4,5], while their inflammatory activation is a prerequisite for elimination of insults (infections or trauma) and tissue recovery [4]. However, aberrant microglial activation can promote neurodegeneration [4,6,7,8,9]. 

Steroid hormones deriving either from the periphery or locally produced in the CNS can affect neuronal and glial function [10,11]. In humans, dehydroepiandrosterone (DHEA) and its sulfate ester are abundant circulating steroid hormones, which are synthesized in the adrenal cortex, the gonads and the brain [11]. Their concentration declines with age, which has been associated with the development of neurologic and neurodegenerative disorders, such as depression and Alzheimer’s disease (AD) [12,13,14,15,16]. Administration of DHEA to animal models of multiple sclerosis (MS) or Parkinson’s disease (PD) had beneficial effects [17,18,19,20,21,22,23], which could be attributed to its neuroprotective [16,24,25,26,27,28,29,30] as well as anti-inflammatory properties [11,16,17,18,31,32,33,34,35,36], including mitigation of microglia-mediated neuroinflammation [18,37,38]. However, since DHEA is converted to androgens and the latter to estrogens, its metabolites might counteract some of its effects [10,18]. Moreover, the increased risk of hormone-related cancer development due to its metabolism to androgens and estrogens hinders its potential clinical use. Therefore, Calogeropoulou et al., 2009, synthesized a series of DHEA analogues with modifications at C3 or C17, which are key positions for the metabolic transformation of DHEA [39]. The most biologically interesting derivative was BNN27, which was shown to exert neuroprotective function, promote axonal growth through synergism with NGF, and restrain microglial inflammation [40,41]. DHEA, as well as its analogue BNN27, were found to bind and activate the nerve growth factor (NGF) receptor, tropomyosin-related kinase A (TRKA), and trigger downstream AKT signaling [28,36,38,40,41]. In accordance, NGF, apart from being a potent neuroprotective agent, also exerts anti-inflammatory effects and promotes amyloid beta (Aβ) peptide clearance in microglia [36,42,43,44]. Hence, targeting TRKA is particularly appealing since this may promote neuroprotection as well as modulate inflammation [45]. Here, we replaced the metabolically labile C-17-spiro oxirane ring in BNN27 with the more stable cyclopropane moiety [46]. In this way, the potential epoxide ring-opening that can result in activity loss may be avoided [47]. Thereby we synthesized the congener compound of BNN27, ΕΝΤ-A010, and show that it demonstrates neuroprotective properties and modulates microglial function in a TRKA-dependent manner. 

## 2. Materials and Methods

### 2.1. Synthesis of ENT-A010

Reactions were run in flame-dried glassware under an atmosphere of argon or nitrogen. All solvents were dried and/or purified according to standard procedures prior to use. Melting points were determined with an Electrothermal Digital Melting Point Apparatus, Cole-Parlmer ET0001/Version 1.0, and were uncorrected. Optical rotations were measured with a P3000 series polarimeter (Krüss Optronic, Hamburg, Germany). NMR spectra were recorded on Varian spectrometers (Varian, Palo Alto, CA, USA). ^1^H NMR spectra were recorded at 300 MHz or 600 MHz, ^13^C NMR spectra were recorded at 75 MHz or 150 MHz and were internally referenced to residual solvent peaks. Chemical shifts are reported in *δ* units, parts per million (ppm). Low-resolution mass spectra were recorded on a LC-MSn Fleet mass spectrometer (Thermo Scientific, Waltham, MA, USA) using MeOH as solvent. High-resolution mass spectrometry (HRMS) spectra were recorded on UPLC-MSn Orbitrap Velos mass spectrometer (Thermo Scientific). Flash column chromatography (FCC) was performed on silica gel 60 (230–400 mesh, Merck, Darmstadt, Germany) and thin-layer chromatography (TLC) on pre-coated glass plates 60 F254 (0.2 mm, Merck). Spots were visualized with UV light at 254 nm and phosphomolybdic acid stain (PMA, 10% in absolute ethanol). The purity of ENT-A010 was determined by high-performance liquid chromatography (HPLC) using Nucelosil 100-5 C18 HD column, 5 μm (4.6 × 250 mm), flow rate 1 mL/min, eluting with H_2_O-CH_3_CN gradient employing UV detection at 254 nm, retention time *t_R_* = 15.713 min. Gradient information: 0.0–8.0 min ramped from 50% H_2_O—50% CH_3_CN to 100% CH_3_CN; 8.0–13.0 min held at 100% CH_3_CN; 13.0–25 min returned to 50% H_2_O–50% CH_3_CN (Supplementary Figure S1). The purity of ENT-A010 was 97.25%.

#### 2.1.1. Synthesis of (E)-(3β-hydroxy-5-androsten-17-ylidene)ethyl ester (1)

To a solution of DHEA (2.0 g, 6.94 mmol) and triethyl phosphonoacetate (15.46 mL, 77.9 mmol) in anhydrous tetrahydrofuran (THF)/absolute ethanol 1:1 (21.4/21.4 mL) was added dropwise at room temperature (RT), a solution of sodium ethoxide (EtONa) in absolute ethanol (prepared from 1.59 g Na in 30 mL ethanol). The resulting mixture was refluxed overnight. Subsequently, the reaction was cooled to 0 °C and was carefully quenched with water and acidified with 10% aq. HCl until completion of the formation of a precipitate. The solid was filtered under reduced pressure, washed with water (30 mL × three times) and petroleum ether 40–60 °C (30 mL × 3 times), and air-dried. The solid was dissolved in CH_2_Cl_2_ and the solution was dried over anhydrous Na_2_SO_4_, filtered and the filtrate was evaporated under reduced pressure to afford compound 1 (2.39 g, 96% yield) as a pure white crystalline solid. The compound was used in the next step without further purification. 

Mp: 178–180 °C; ${\left[a\right]}_{D}^{25}$ = −78° (c = 0.006 g/mL, CHCl_3_); R_F_: 0.5 (petroleum ether 40–60 °C/acetone 80:20); ^1^H NMR (600 MHz, CDCl_3_): *δ* 0.84 (s, 3H), 1.03 (s, 3H), 1.28 (t, *J*= 7.1 Hz, 3H, OCH_2_CH_3_), 1.31–2.34 (m, 18H), 2.78–2.90 (m, 2H), 3.48–3.57 (m, 1H, 3*α*-H), 4.15 (q, *J* = 7.1 Hz, 2H, OCH_2_CH_3_), 5.36 (d, *J* = 5.0 Hz, 1H, 6-H), 5.55 (bs, 1H, 20-H); ^13^C NMR (150 MHz, CDCl_3_): *δ* 14.5, 18.4, 19.6, 21.1, 24.6, 30.6, 31.7, 31.8, 35.3, 36.8, 37.4, 42.4, 46.2, 50.4, 54.0, 59.7, 71.7, 71.8, 108.8, 121.5, 140.9, 167.6, 176.3; ESI-HRMS: *m*/*z* [M + H]^+^ calculated for C_23_H_35_O_3_ 359.2580, found 359.2581; *m*/*z* [M + Na]^+^ calculated for C_23_H_34_O_3_Na [M+Na]^+^ 381.2399, found 381.2400.

#### 2.1.2. Synthesis of (E)-[3β-(t-butyldimethylsilyloxy)-5-androsten-17-ylidene]ethyl ester (2)

To a solution of compound 1 (2.25 g, 6.28 mmol) in anhydrous THF (20 mL), imidazole (1.33 g, 19.5 mmol) and iodine (4.76 g, 37.5 mmol) were added at 0 °C and the mixture was stirred at 0 °C for 30 min. Subsequently, *tert*-butyldimethylsilyl chloride (1.05 g, 6.67 mmol) was added and the resulting mixture was stirred at 25 °C overnight. After completion of the reaction, the solvent was evaporated in vacuo and the residue was diluted and extracted with ethyl acetate (EtOAc). The organic layer was washed with sat. aq. Na_2_S_2_O_4_, brine and dried over anhydrous Na_2_SO_4_. The solvent was removed in vacuo and the residue was purified by FCC (elution solvent: petroleum ether 40–60 °C/ethyl acetate 95:5) to afford compound 2 as a white solid (2.36 g, 93% yield). 

Mp: 100–102 °C; ${\left[a\right]}_{D}^{25}$ = −50° (c = 0.0056 g/mL, CHCl_3_); R_F_: 0.86 (petroleum ether 40–60 °C/EtOAc 80:20); ^1^H NMR (600 MHz, CDCl_3_): *δ* 0.05 (s, 6H, Si(CH_3_)_2_), 0.83 (s, 3H), 0.89 (s, 9H, C(CH_3_)_3_), 1.02 (s, 3H), 1.28 (t, *J* = 7.1 Hz, 3H, OCH_2_CH_3_), 1.32–2.31 (m, 17H), 2.79–2.89 (m, 2H) 3.42–3.54 (m, 1H, 3*α*-H), 4.15 (q, *J* = 7.1 Hz, 2H, OCH_2_CH_3_), 5.33 (d, *J* = 5.3 Hz, 1H, 6-H), 5.54 (t, *J* = 2.3 Hz, 1H, 20-H); ^13^C NMR (75 MHz, CDCl_3_): *δ* −4.3, 14.5, 18.4, 19.6, 21.1, 24.6, 26.1, 30.6, 31.7, 31.8, 32.2, 35.4, 36.8, 37.5, 42.9, 46.2, 50.5, 54.0, 59.7, 72.7, 108.8, 120.9, 121.0, 141.8, 167.6, 176.4; ESI-HRMS: *m*/*z* [M + H]^+^ calculated for C_29_H_49_O_3_Si 473.3445, found 473.3445; *m*/*z* [M + Na]^+^ calculated for C_29_H_48_O_3_SiNa [M + Na]^+^ 495.3263, found 495.3265.

#### 2.1.3. Synthesis of (E)-3β-(t-butyldimethylsilyloxy)-pregna-5,17(20)-dien-21-ol (3)

To a solution of compound 2 (2.20 g, 4.65 mmol) in anhydrous ΤHF (94 mL) was added dropwise at −78 °C diisobutylaluminum hydride (DIBAL-H) [(1.0 M in hexane), 18.7 mL, 18.7 mmol]. The reaction mixture was stirred at −78 °C for 2.5 h and at 25 °C for an additional 1 h. After completion of the reaction, saturated aqueous NH_4_Cl (40 mL) was added at 0 °C and the solvent was evaporated in vacuo. EtOAc (30 mL) was added to the residue and the organic layer was washed with 10% aq. HCl (20 mL) and brine (40 mL), dried over anhydrous Na_2_SO_4_, filtered and the filtrate evaporated under reduced pressure to afford compound 3 as a white pure crystalline solid (2.0 g, quantitative yield). Compound 3 was used in the next step without further purification.

Mp: 129–131 °C; ${\left[a\right]}_{D}^{25}$ = −39° (c = 0.0054 g/mL, CHCl_3_); R_F_: 0.46, (petroleum ether 40–60 °C/EtOAc 80:20); ^1^H NMR (600 MHz, CDCl_3_): *δ* 0.06 (s, 6H, Si(CH_3_)_2_), 0.78 (s, 3H), 0.89 (s, 9H, C(CH_3_)_3_), 1.02 (s, 3H), 1.19–2.43 (m, 18H), 3.43–3.53 (m, 1H, 3*α*-H), 4.09 (dd, *J* = 12.2, 6.3 Hz, 1H, 21-H), 4.15 (dd, *J* = 12.0, 7.4 Hz, 1H, 21-H) 5.25 (t, *J* = 6.7 Hz, 1H, 20-H), 5.32 (d, *J* = 5.1 Hz, 1H, 6-H); ^13^C NMR (75 MHz, CDCl_3_): *δ* −4.4, 14.3, 18.4, 18.7, 19.6, 21.1, 21.2, 24.5, 26.1, 26.3, 31.8, 31.9, 32.2, 35.8, 36.9, 37.5, 42.9, 44.0, 50.7, 72.7, 115.7, 121.0, 141.8, 155.9; ESI-HRMS: *m*/*z* [M + Na]^+^ calculated for C_27_H_46_O_2_SiNa 453.31584, found 453.3158.

#### 2.1.4. Synthesis of (17S,20S)-3β-(t-butyldimethylsilyloxy)-17α,20-methan-5-pregnane-21-ol (4)

To a solution of compound 3 (1.8 g, 4.17 mmol) in anhydrous toluene (33.6 mL) was added at 25 °C diiodomethane (CH_2_I_2_) (1.8 mL, 21.6 mmol). The reaction mixture was then cooled to −78 °C and a solution of diethylzinc (0.9 M in hexane, 24 mL, 21.6 mmol) was added and the reaction mixture was stirred at 25 °C for 2 h. After completion of the reaction (monitored by ^1^H NMR), the reaction mixture was quenched with 10% aqueous HCl at 0 °C until pH 5.5, followed by extraction with EtOAc (30 mL × 3 times). The organic layer was washed with brine, dried over anhydrous Na_2_SO_4_ and the solvent was removed in vacuo. The residue was purified by FCC (petroleum ether 40–60 °C/EtOAc 90/10 →85/15) to obtain compound 4 (1.1 g, 60% yield). 

Mp: 154–157 °C; ${\left[a\right]}_{D}^{25}$ = −50° (c = 0.0036 g/mL, CHCl_3_); R_F_: 0.46 (petroleum ether 40–60 °C/EtOAc 80:20); ^1^H NMR (600 MHz, CDCl_3_): *δ* 0.06 (s, 6H, Si(CH_3_)_2_), 0.78 (s, 3H), 0.89 (s, 9H, C(CH_3_)_3_), 0.90–0.97 (m, 1H) 1.01 (s, 3H), 1.03–2.32 (m, 20H), 3.45–3.54 (m, 2H), 3.58–3.66 (m, 1H, 22-H), 5.33 (d, *J* = 5.3 Hz, 1H, 6-H); ^13^C NMR (75 MHz, CDCl_3_): *δ* −4.4, 16.3, 17.1, 18.4, 19.6, 19.8, 20.6, 25.2, 26.1, 29.1, 32.1, 32.2, 32.7, 33.3, 36.3, 36.8, 37.5, 41.1, 42.9, 50.6, 54.9, 65.3, 72.7, 121.1, 141.7; ESI-HRMS: *m*/*z* [M + Na]^+^ calculated for C_28_H_48_O_2_SiNa 467.3314, found 467.3314.

#### 2.1.5. Synthesis of (17S,20S)-3β-(t-butyldimethylsilyloxy)-17α,20-methan-5-pregnane-21-al (5)

To a solution of compound 4 (0.85 g, 1.91 mmol) in dry CH_2_Cl_2_ (100 mL) was added at 0 °C Dess-Martin periodinane (1.62 g, 3.82 mmol) and the reaction mixture was stirred at 25 °C for 1.5 h. After completion of the reaction (checked by TLC), a mixture of saturated aqueous NaHCO_3_ and 10% aq. Na_2_S_2_O_4_ (1:1) were added and the resulting mixture was stirred for 30 min. The reaction mixture was extracted with diethyl ether (Et_2_O) (30 mL × 3 times) and the combined organic layers washed with saturated aqueous NaHCO_3_ and brine, dried over Na_2_SO_4_ and the solvent was removed in vacuo. The residue was purified by FCC (petroleum ether 40–60 °C/EtOAc 98/2→96/4) to afford compound 5 as a white crystalline solid (0.71 g, yield = 84%). Mp: 114–116 °C; ${\left[a\right]}_{D}^{25}$ = −21° (c = 0.0056 g/mL, CHCl_3_); R_F_: 0.78 (petroleum ether 40–60 °C/EtOAc 80:20); ^1^H NMR (600 MHz, CDCl_3_): *δ* 0.05 (s, 6H, Si(CH_3_)_2_), 0.80 (s, 3H), 0.88 (s, 9H, C(CH_3_)_3_), 0.92–0.99 (m, 1H), 1.00 (s, 3H), 1.04–2.30 (m, 21H), 3.42–3.52 (m, 1H, 3*α*-H), 5.33 (d, *J* = 5.1 Hz, 1H, 6-H), 9.08 (d, *J* = 6.2 Hz, 1H, *CHO*); ^13^C NMR (75 MHz, CDCl_3_): *δ* −4.4, 17.1, 18.4, 19.6, 20.5, 21.1, 25.3, 26.0, 29.6, 31.0, 32.0, 32.1, 32.6, 33.1, 36.8, 37.5, 42.3, 42.9, 43.8, 50.4, 53.9, 72.6, 120.9, 141.7, 202.0; ESI-HRMS: *m*/*z* [M + Na]^+^ calculated for C_28_H_46_O_2_SiNa 465.31638, found 465.3164.

#### 2.1.6. Synthesis of (17S,20S)-3β-(t-butyldimethylsilyloxy)-17α,20-Methan-5,21-pregna-dien-22-ethyl ester (6)

To a suspension of NaH 60% in mineral oil (0.3 g, 6.5 mmol) in dry THF (4 mL) was added at 0 °C triethylphosphonoacetate (1.3 mL, 6.5 mmol) and the reaction mixture was stirred at RT for 30 min. Subsequently, a solution of compound 5 (0.72 g, 1.62 mmol) in dry THF (16.4 mL) was added at 0 °C and the reaction mixture was stirred at the same temperature for 30 min. The reaction was quenched with saturated aqueous NH_4_Cl and extracted with EtOAc (20 mL × 3 times). The combined organic layers were washed with brine, dried over anhydrous Na_2_SO_4_ and the solvent was removed in vacuo. The residue was purified by FCC (petroleum ether 40–60 °C/ethyl acetate 99/1→98/2) to afford compound 6 as a white crystalline solid (0.8 g, 96% yield). Mp: 127–129 °C; ${\left[a\right]}_{D}^{25}$ = +30° (c = 0.003 g/mL, CHCl_3_); R_F_: 0.71 (petroleum ether 40–60 °C/EtOAc 90:10); ^1^H NMR (600 MHz, CDCl_3_): *δ* 0.06 (s, 6H, Si(CH_3_)_2_), 0.50 (t, *J* = 4.9 Hz, 1H, 21-H), 0.75 (s, 3H), 0.89 (s, 9H, C(CH_3_)_3_), 1.00 (s, 3H), 1.28 (t, *J* = 7.2 Hz, 3H, CH_2_CH_3_), 1.36–2.31 (m, 21H), 3.45–3.53 (m, 1H, 3*α*-H), 4.14–4.20 (m, 2H, CH_2_CH_3_), 5.35 (d, *J* = 3.0 Hz, 1H, 6-H), 5.85 (d, *J* = 15.4 Hz, 1H, 23-H), 6.61 (dd, *J* = 15.3, 10.4 Hz, 1H, 22-H); ^13^C NMR (150 MHz, CDCl_3_): *δ* −4.4, 14.5, 16.7, 18.4, 19.6, 20.6, 22.0, 22.5, 25.2, 26.1, 29.9, 32.1, 32.2, 32.6, 33.4, 36.9, 37.6, 41.7, 42.1, 42.9, 50.5, 54.7, 60.1, 72.7, 118.9, 121.1, 141.7, 152.9, 166.9; ESI-HRMS: *m*/*z* [M + H]^+^ calculated for C_32_H_53_O_3_Si 513.3758, found 513.3757; *m*/*z* [M + Na]^+^ calculated for C_32_H_53_O_3_SiNa 535.3578, found 535.3576.

#### 2.1.7. Synthesis of (17S,20S)-3β-hydroxy-17α,20-methan-5,21-pregna-dien-22-ethyl ester (ENT-A010)

To a solution of compound 6 (0.8 g, 1.56 mmol) in anhydrous THF (47 mL) TBAF (1.0 M in THF) (5.4 mL, 5.4 mmol) was added dropwise at 0 °C and the reaction mixture was stirred at RT overnight. The reaction was quenched with water at 0 °C, and the resulting mixture was extracted with EtOAc (30 mL × 3 times). The organic layer was washed with brine, dried over anhydrous Na_2_SO_4_ and the solvent was removed in vacuo. The residue was purified by FCC (elution solvent: Hexane/ethyl acetate 80/20) to afford compound ENT-A010 as a white crystalline solid (0.62 g, quantitative yield). Mp: 129–132 °C; ${\left[a\right]}_{D}^{25}$ = +39° (c = 0.0036 g/mL, CHCl_3_); R_F_: 0.20 (hexane/EtOAc 80:20); ^1^H NMR (600 MHz, CDCl_3_): *δ* 0.50 (t, *J* = 4.9 Hz, 1H, 21-H), 0.76 (s, 3H), 0.91–0.98 (m, 1H), 1.01 (s, 3H), 1.05–1.15 (m, 2H), 1.28 (t, *J* = 7.2 Hz, 3H, CH_2_CH_3_), 1.36–2.34 (m, 18H), 3.46–3.56 (m, 1H, 3*α*-H), 4.11–4.21 (m, 2H, CH_2_CH_3_), 5.36 (d, *J* = 4.8 Hz, 1H, 6-H), 5.86 (d, *J* = 15.4 Hz, 1H, 23-H), 6.61 (dd, *J* = 15.4, 10.4 Hz, 1H, 22-H); ^13^C NMR (75 MHz, CDCl_3_): *δ* 14.5, 16.7, 19.5, 20.6, 21.9, 22.4, 25.2, 29.8, 31.7, 32.0, 32.6, 33.4, 36.7, 37.4, 41.6, 42.1, 42.4, 50.4, 54.6, 60.1, 71.8, 118.9, 121.6, 140.9, 152.8, 166.9; APCI-HRMS: *m*/*z* [M + H]^+^ calculated for C_26_H_39_O_3_ 399.28892, found 399.2889. 

### 2.2. Mice 

Eight- to twelve-week-old C57BL/6J male mice were used (Charles River Laboratories, Sulzfeld, Germany). Mice had free access to food and water and were housed under a 12-h light-dark cycle. They were intraperitoneally (i.p.) injected at two consecutive days with freshly prepared ENT-A010 (70 mg/kg) in phosphate-buffered saline (PBS) with 4.5% ethanol and 1% bovine serum albumin (BSA) (Sigma-Aldrich, St. Louis, MO, USA), or vehicle control solution. One hour after the second injection, the mice were injected i.p. with lipopolysaccharide (3 mg/kg) (Ultrapure LPS, *Escherichia coli* 0111:B4, Invivogen, San Diego, CA, USA). After 16 h, they were deeply anesthetized with ketamine/xylazine and intracardially (i.c.) perfused with PBS. Afterward, brain regions were isolated and snap-frozen for further analyses, or brains were collected for histological analyses. For the study of ENT-A010 brain uptake, mice were administered i.p. 70 mg/kg ENT-A010 (in PBS containing 4.5% ethanol and 1% BSA) or vehicle solution and they were euthanized 1 and 2 h after injection with ENT-A010 and 1.5 h after injection with control solution. Different brain regions (hippocampus, hypothalamus, cortex, brainstem, cerebellum), livers and spleens were collected and snap-frozen. Animal experiments were approved by the Landesdirektion, Dresden, Germany.

### 2.3. ENT-A010 Detection in Tissues

Tissues were weighed and homogenized in 300 μL ice-cold distilled H_2_O/methanol solution (25/75 *v*/*v*) and sonicated for 20 min. Three volumes of ice-cold acetonitrile were added, followed by sonication for 10 min and centrifugation at 14,000× *g*/15 min/4 °C. Next, 100 μL of ice-cold acetonitrile were added to the supernatant, followed again by a 10 min-sonication and centrifugation at 14,000× *g*/15 min/4 °C. The supernatant was vacuum-dried at a SpeedVac without heating and samples were stored at −80 °C. The analysis was performed on an LTQ-Orbitrap Velos mass spectrometer (MS) (Thermo Fisher Scientific, Bremen, Germany) connected to an Accela ultra-high-performance LC (UHPLC) system. An Acquity UPLC BEH C18 VanGuard pre-column (130 Å, 1.7 μm, 2.1 mm × 100 mm) coupled to an Acquity UPLC BEH C18 column (130 Å, 1.7 μm, 2.1 mm × 5 mm) were used. To monitor over time the instruments’ performance and chromatographic integrity, including retention time-shifts, quality control samples were prepared at three concentrations, 4 ng/mL, 70 ng/mL and 500 ng/mL. 

Deuterated pregnenolone (pregnenolone 17,21,21,21-D4) at 70 ng/mL served as an internal standard. Monitoring occurred in positive ion mode. The standard curve concentration range was 1–2000 ng/mL (Y = 0.000265415∗X; R^2^ = 0.9960; W:1/x). The injection volume was set at 5 μL, and the mobile phase flow rate was set at 0.2 mL/min. Mobile phase solvents were A (95% H_2_O, 5% methanol, 0.1% formic acid) and B (methanol, 0.1% formic acid). The eluting gradient program was the following: 0–0.1 min (40% A, 60% B), 0.1–0.5 min (20% A, 80% B), 0.6–6.5 min (5% A, 95% B), 6.51–8.0 min (40% A, 60% B). Data were processed with Xcalibur software (version 2.1, Thermo Scientific, Waltham, MA, USA).

### 2.4. Cell Isolation and Culture

Primary dorsal root ganglia (DRG) neurons were isolated from P0-P1 mouse pups (C57BL/6J, Jackson Laboratory, Bar Harbor, ME USA) according to a previously described protocol [48]. Briefly, spinal cords from P0-P1 pups were dissected and DRG were isolated in ice-cold Hank’s Balanced Salt Solution (HBSS). Axons were removed from the ganglia. Ganglia were then treated with 0.25% Trypsin for 15 min at 37 °C, washed three times with HBSS and three times with Dulbecco′s Modified Eagle′s Medium (DMEM) containing 10% FBS. DRG were dissociated using fire-polished glass Pasteur pipettes, counted and plated in DMEM with 10% FBS and 1% penicillin/Streptomycin medium. Two hours later, the medium was changed to neurobasal medium (Gibco, Grand Island, New York, NY, USA) containing 2% B27 supplement (Gibco, Grand Island, New York, NY, USA), 100 ng/mL NGF (N-100, Alomone Labs, Jerusalem, Israel), 1X GlutaMax (Gibco, Grand Island, New York, NY, USA), 10 mM 2-Hydroxyethylpiperazine-N′-2-ethanesulfonic acid (HEPES, Gibco, Grand Island, New York, NY, USA), 1% penicillin/streptomycin (Gibco, Grand Island, New York, NY, USA) and 10 μM 5-fluoro-2′-deoxyuridine (Calbiochem, Darmstadt, Germany). DRG neurons were cultured at 37 °C and 5% CO_2_. 

Primary hippocampal neurons were isolated (C57BL/6J, Jackson Laboratory) as previously described [49]. Briefly, the brains of E17.5 mouse embryos were dissected and submerged in ice-cold HBSS. Hippocampi were removed and treated with 0.25% Trypsin for 15 min at 37 °C. Then, they were washed three times with HBSS and three times with DMEM medium containing 10% FBS and 1% penicillin/streptomycin. Afterward, they were dissociated using fire-polished glass Pasteur pipettes, counted and plated in DMEM with 10% FBS and 1% penicillin/streptomycin medium. The medium was changed to neurobasal medium containing 2% B27, 1X GlutaMax, 10 mM HEPES, 2 μM cytosine β-D-arabinofuranoside (C1768, Sigma-Aldrich) and 1% penicillin/streptomycin 2 h after plating. Cells were cultured at 37 °C and 5% CO_2_.

Primary microglia were isolated as previously described [38,43]. Briefly, brains of 8–12 week old C57BL/6J male mice were digested with a papain (Sigma-Aldrich, St. Louis, MO, USA)- and dispase (Sigma-Aldrich, St. Louis, MO, USA)-containing solution. Single-cell suspensions were seeded onto culture flasks coated with poly-L-lysine (PLL) (Sigma-Aldrich, St. Louis, MO, USA) and maintained in DMEM/F12 supplemented with 10% fetal bovine serum (FBS), 1% penicillin/streptomycin and 7 ng/mL recombinant murine granulocyte and macrophage colony-stimulating factor (GM-CSF) (PeproTech, East Windsor, NJ, USA) at 37 °C and 5% CO_2_. Three weeks after isolation, floating cells were collected 3 times a week and seeded on PLL-coated 24-well plates at 2 × 10^5^ cells/well density for RNA extraction, 2.5 × 10^5^ cells/well in 12-well plates for phagocytosis assays and 5 × 10^5^ cells/well in six-well plates for western blot (WB). Cells were cultured without GM-CSF for 2 days prior to treatments. All treatments were performed in DMEM/F12 supplemented with 1% charcoal-stripped FBS and 1% penicillin/streptomycin at 37 °C and 5% CO_2_.

PC12 cells (obtained from LGC, the European partner of ATCC) were grown in DMEM medium (11965084, Gibco, Grand Island, NY, USA) containing 10% horse serum (Gibco, Grand Island, NY, USA), 5% FBS (Gibco), 1% penicillin/streptomycin (Gibco) at 37 °C and 5% CO_2_. 

BV2 cells were obtained from Interlab Cell Line Collection (ICLC, Genova, Italy) and maintained in RPMI-1640 medium supplemented with 10% FBS and 1% penicillin/streptomycin at 37 °C and 5% CO_2_. 

### 2.5. TUNEL Assay

DRG neurons were maintained for 48 h without NGF and in the presence of a NGF-neutralizing antibody (N8773, 1:500, Sigma-Aldrich, St. Louis, MO, USA) with ENT-A010 (500 nM) or the same amount of dimethyl sulfoxide (DMSO). Primary hippocampal neurons were treated for 48 h with 5 µM oligomeric Aβ (AnaSpec, Fremont, CA, USA) in the presence of ENT-A010 (500 nM, newly supplemented every 24 h) or vehicle control (DMSO). Cells were then fixed with 4% paraformaldehyde (PFA) and labelled with terminal deoxynucleotidyl transferase dUTP nick-end labeling (TUNEL, Roche, Hertfordshire, UK) following the manufacturer’s protocol. Subsequently, cells were immunostained against TUJ1 (1:2000, 801201, Biolegend, San Diego, CA, USA) and anti-mouse Cy3 (1:1000, Invitrogen, A10521, Waltham, MA, USA) and imaged with a Leica SP8 confocal microscope. The percentage of apoptotic neurons was determined by normalizing the number of TUNEL^+^ cells to the total number of Hoechst^+^TUJ1^+^ neuronal cells. The FIJI software was used to determine the numbers of TUNEL^+^ and Hoechst^+^ cells [50].

Aβ_1–42_ peptide was prepared according to the manufacturer’s instructions. Oligomeric Aβ, which is considered to be the toxic form of amyloid, was prepared as previously described with slight modifications [51]. Aβ peptide was diluted in DMEM at 5 μM and incubated for 24 h at 37 °C. It was then centrifuged for 5 min at 14,000× *g* and the supernatant containing oligomeric Aβ was collected.

### 2.6. CellTox Assay

PC12 cells were plated in 96-well plates, serum-deprived for 4 h and subsequently treated with or without NGF (100 ng/mL) or ENT-A010 (500 nM) in the presence or absence of GW441756 TRKA inhibitor (20 μM, G-190, Alomone Labs, Jerusalem, Israel) for another 24 h. The CellTox assay (Promega, Leiden, Belgium) was performed according to the manufacturer’s instructions. Hoechst (1:10,000, Invitrogen, MA, USA) was added for 30 min along with CellTox reagent and cells were imaged with a Zeiss AXIO Vert A1 fluorescent microscope. The number of CellTox^+^ (dead) cells was normalized to the number of Hoechst^+^ cells, the latter depicting the total number of cells, per image. The numbers of CellTox^+^ and Hoechst^+^ cells were determined using the FIJI software. 

### 2.7. Synaptophysin Detection

Primary hippocampal neurons were treated for 4 h with 5 μM oligomeric Aβ and ENT-A010 (500 nM) or control vehicle (DMSO). Cells were then fixed with 4% PFA and immunostained against TUJ1, Synaptophysin (1:1000, PA1-1043, Invitrogen, MA, USA) and anti-mouse Alexa fluor 488 (1:1000, A-11029, Invitrogen, MA, USA) or anti-rabbit Alexa fluor 546 (1:1000, A10040, Invitrogen, MA, USA). Images were acquired with a Leica SP8 confocal microscope and the total area of Synaptophysin-positive puncta was measured and normalized to TUJ1 total area using the FIJI software. 

### 2.8. Phagocytosis Assay

Primary microglial cells were treated on two consecutive days with 1 µM ENT-A010 or an equal volume of DMSO. One hour after the second dose, cells were stimulated or not for 24 h with 100 ng/mL LPS (Ultrapure from *E. coli* K12, Invivogen, San Diego, CA, USA). In some experiments, cells were treated with the AKT inhibitor MK2206 (2.5 µM, Cayman Chemical, MI, USA), 30 min prior to the first treatment with ENT-A010. Cells were then given for 2 h 750 nM HiLyte™ Fluor 555-labeled Aβ_1–40_ (Anaspec, Fremont, CA, USA). Afterward, they were harvested by gentle scraping, washed twice with FACS buffer (3% FBS and 2 mM EDTA in PBS), re-suspended in FACS buffer and analyzed with a BD FACSCanto II flow cytometer using the FACSDiva 6.1.3 software. The mean fluorescent intensity was determined after debris and doublet exclusion in a total number of 10,000 events per sample.

### 2.9. Immunoprecipitation 

PC12 cells were serum-deprived for 4 h and subsequently treated with 100 ng/mL NGF, 500 nM ENT-A010 or vehicle for 30 min. Cells were then lysed in Pierce™ IP Lysis Buffer (Thermo Scientific, Rockford, IL, USA) containing protease (Calbiochem, Darmstadt, Germany) and phosphatase inhibitors (Calbiochem, Darmstadt, Germany). Lysates were immunoprecipitated overnight at 4 °C with anti-TRKA (1:100, 06-574, Sigma-Aldrich, St. Louis, MO, USA) followed by 4 h of incubation with protein G-plus agarose beads (sc-2002, Santa Cruz Biotechnology, CA, USA). Beads were then collected, washed 3 times with lysis buffer, re-suspended in SDS loading buffer and subjected to WB against phosphorylated tyrosine (1:1000, BAM1676, R&D Systems, Minneapolis, MN, USA). 

### 2.10. Western Blotting

Proteins extracts were prepared in ice-cold RIPA buffer supplemented with protease and phosphatase inhibitors (Roche, Hertfordshire, UK). Protein concentration was determined with the BCA assay (Thermo Scientific, Rockford, IL, USA). Protein lysates were mixed with reducing Laemmli SDS sample buffer (Alfa Aesar, Haverhill, MA, USA), denatured at 95 °C for 5 min and 100 µg of protein were loaded on a polyacrylamide gel and separated with SDS-PAGE. Afterward, proteins were transferred onto nitrocellulose membranes and blocking was performed with 5% BSA in TBS-T buffer overnight at 4 °C. The next day, primary antibodies were added to the membranes and incubated overnight at 4 °C. Primary antibodies used were following: anti-phosphoTRKA (phospho Y490) (Abcam, ab1445), anti-TRKA (Abcam, ab76291), anti-phosphoAKT (Ser473) (Cell Signaling, #4060), anti-AKT (Cell Signaling, #4691) and anti-Vinculin (Cell Signaling, #4650), all diluted at 1:1000 in 5% BSA in TBS-T. On the third day, goat anti-rabbit IgG horseradish peroxidase-conjugated antibody (1:3000, R&D Systems, Minneapolis, MN, USA) was added to the membranes and incubated for 2 h at RT. Finally, membranes were washed with TBS-T and developed using SuperSignal West Pico Chemiluminescent Substrate (Life Technologies, Carlsbad, CA, USA) or SuperSignal West Fempto Chemiluminescent Substrate (Life Technologies) and the luminescent image analyzer LAS-3000 (Fujifilm, Dusseldorf, Germany). The intensity of the bands was quantified using the FIJI software [38,43].

### 2.11. Immunofluorescent Staining and Confocal Microscopy

Brains were post-fixed with 4% PFA in PBS for 4 h at 4 °C, immersed in a 30% sucrose solution in PBS and incubated overnight at 4 °C. Then, the tissues were embedded in OCT compound (Tissue-Tek), and frozen at −80 °C. Fourteen-µm-thick coronal sections were cut by cryosectioning and transferred onto slides. Antigen retrieval was performed in 0.1 M citrate buffer (pH 6.0) for 10 min in a pressure cooker. Tissue sections were blocked with a serum-free Protein Block (Dako, Denmark) for 2 h at RT, followed by overnight incubation with anti-IBA1 primary antibody (019-19741, Wako, Osaka, Japan) diluted 1:750 in antibody diluent (Dako, Denmark). The next day, sections were washed and incubated with donkey anti-rabbit IgG-Alexa Fluor 555 (Life Technologies, Carlsbad, CA, USA) diluted 1:350 in antibody diluent for 2 h. Then, the nuclei were counterstained for 5 min with DAPI (1:10,000 in PBS) and the slides were mounted using Fluoromount (Life technologies, Carlsbad, CA, USA).

Confocal imaging was performed using a Zeiss LSM880 system (Zeiss, Jena, Germany). Images were acquired with the ZEN 3.2 blue edition software. Sections were excited with two laser lines at 405 nm, and 561 nm and emissions were detected with photomultiplier tube detectors within a window of 415–478 nm (DAPI) and 571 nm-625 nm (Alexa-Fluor 555), respectively. An air immersion 20×/0.8NA Plan-Apochromat was used at zoom factor 1. Pixel size corresponded to 210 nm × 210 nm. Laser power, pinhole size and photomultiplier gain were determined for each fluorophore at the beginning and were kept constant throughout the imaging procedure. Six Z-stacks were taken with a step size of 1.57 μm, through a total thickness of 7.868 μm per section. Processing was performed with the FIJI software.

### 2.12. RNA Extraction and qRT-PCR

Total RNA was extracted from cells or tissues using the Nucleospin RNA isolation kit (Macherey-Nagel, Dueren, Germany), according to the manufacturer’s instructions. cDNA was synthesized using the iScript cDNA synthesis kit (Bio-Rad, Hercules, CA, USA). qPCR was performed using the SsoFast Eva Green Supermix (Bio-Rad Hercules, CA, USA), a CFX384 real-time System C1000 Thermal Cycler (Bio-Rad), and the Bio-Rad CFX Manager 3.1 software, as previously described [36,38,43]. The relative amount of mRNA was calculated with the ΔΔCt method, using *18s* as a housekeeping gene. The primer sequences are listed in Table S1.

### 2.13. Statistical Analysis

All values are expressed as the mean ± SEM. Mann-Whitney U or Student’s *t*-test was used for the comparison of two groups. One-way ANOVA followed by Tukey’s multiple-comparison test was used for multiple group comparisons. A *p* < 0.05 was considered to mark statistical significance. Statistical analysis was performed using GraphPad Prism 7 (GraphPad Software Inc., San Diego, CA, USA). 

## 3. Results

### 3.1. Synthesis of ENT-A010 

**Figure 1:**
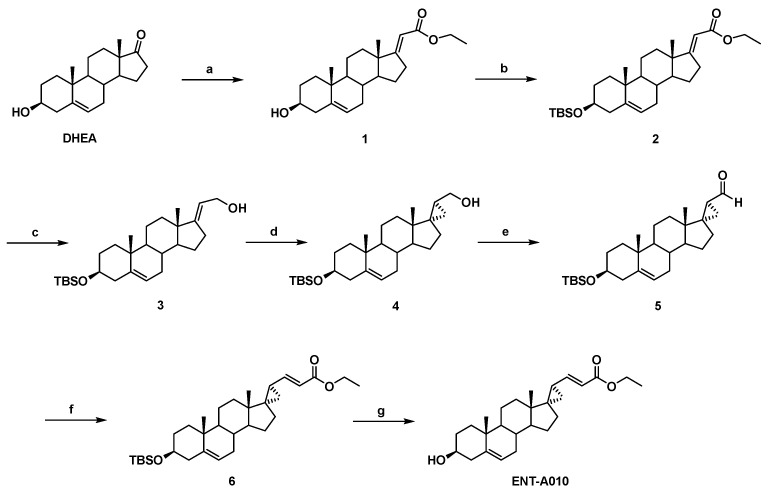
Synthesis of ENT-A010. Reagents and conditions: (**a**) (CH_3_CH_2_O)_2_P(O)CH_2_C(O)OCH_2_CH_3_, EtONa, THF/EtOH 1:1, reflux, overnight; (**b**) TBSCl, Imidazole, I_2,_ THF, 0 °C to 25 °C, overnight; (**c**) DIBAL-H, CH_2_Cl_2_, −78 °C, 2.5 h; (**d**) CH_2_I_2_, Et_2_Zn, −78 °C to 25 °C, 1 h; (**e**) Dess-Martin periodinane, DCM, 0 °C to 25 °C, 1.5 h; (**f**) (CH_3_CH_2_O)_2_P(O)CH_2_C(O)OCH_2_CH_3_, NaH, THF, 0 °C to 25 °C, 0.5 h; (**g**) TBAF (1.0 M in THF), THF, 0 °C to 25 °C, overnight.

The synthesis of ENT-A010 starting from DHEA involves seven high-yielding steps as shown in Figure 1. The Horner–Emmons reaction of DHEA with triethyl phosphonoacetate in the presence of EtONa as a base gave the (*E*)-*a*,*b*-unsaturated ester 1 in 96% yield, which was, in turn, reacted with *tert*-butyldimethylsilyl chloride (TBSCl) to afford the *tert*-butyldimethylsilyl-protected alcohol 2 in 93% yield. Reduction of the ester group in 2 using DIBAL-H gave the allylic alcohol 3 in quantitative yield, which was subjected to a Simmons-Smith cyclopropanation reaction in the presence of CH_2_I_2_ and Et_2_Zn to yield the (17*S*,20*S*)-cyclopropyl derivative 4 in 60% yield [52]. Compound 4 was consequently oxidised with Dess-Martin periodinane in dichloromethane to afford the corresponding aldehyde 5 in 84% yield. Horner-Emmons reaction of aldehyde 5 with triethylphosphonoacetate in the presence of NaH afforded only the *E* isomer of the *a*,*b*-unsaturated ester 6 in 96% yield. Deprotection of the C3 alcohol using tetrabutylammonium fluoride (TBAF) 1.0 M in THF yielded quantitatively ENT-A010. 

### 3.2. ENT-A010 Promotes Neuronal Survival in a TRKA-Dependent Manner

**Figure 2:**
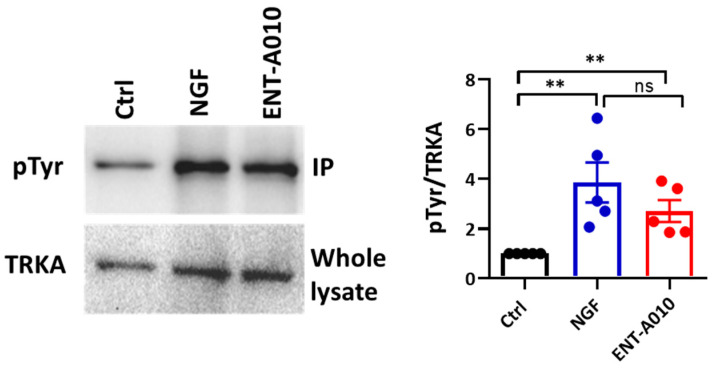
ENT-A010 induced TRKA phosphorylation. PC12 cells were treated for 30 min with NGF (100 ng/mL), ENT-A010 (500 nM), or vehicle control. TRKA was immunoprecipitated, and membranes were immunoblotted for phosphor-Tyrosine (pTyr). Whole-cell lysates were analyzed for TRKA. Representative blots are shown. The intensity of the bands was measured, the ratio pTyr/TRKA was calculated and set in each experiment as 1. Data are shown as mean ± SEM, **: *p* < 0.01; *n* = 5 independent experiments; ns: non-significant.

ENT-A010 was selected for further investigation from a library of novel synthetic C17-spiro-DHEA derivatives (a manuscript describing their synthesis is in preparation) based on its capacity to induce TRKA phosphorylation. To test TRKA phosphorylation, PC12 cells were stimulated for 30 min with ENT-A010 or NGF, and proteins were immunoprecipitated for TRKA and immunoblotted against phosphorylated tyrosine. ENT-A010 induced TRKA phosphorylation, at levels similar to those of NGF (Figure 2). 

**Figure 3:**
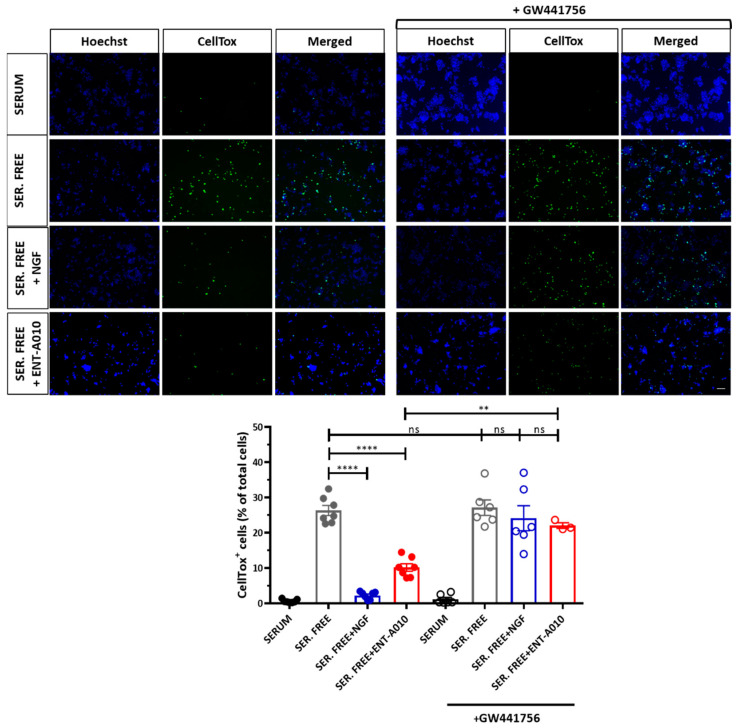
ENT-A010 promoted survival of PC12 cells via TRKA. PC12 cells were treated for 24 h with NGF (100 ng/mL), ENT-A010 (500 nM) or vehicle control, with or without the selective TRKA inhibitor GW174456 under serum starvation. Upper panel: Cells were stained with CellTox (green) and Hoechst (blue). Scale bar: 200 μm (applies to all photos). Lower panel: Quantification of cell viability, calculated as percentage of dead (CellTox^+^) cells per total number of Hoechst^+^ cells in each image. Data are shown as mean ± SEM, *n* = 3–7, **: *p* < 0.01, ****: *p* < 0.0001, ns: non-significant.

Then, we asked if ENT-A010, similarly to DHEA and NGF, may exert neuroprotective function [25,26,28,40,53]. To examine this, we used three different in vitro experimental models to induce cell apoptosis: PC12 cells cultured in serum-deprived conditions, NGF-deprived dorsal root ganglia (DRG) neurons and Aβ-induced cell death of hippocampal neurons. PC12 cells were cultured for 24 h in serum-free medium to undergo apoptosis and ultimately cell death [25,26,54], and treated or not with NGF or ENT-A010 for 24 h. ENT-A010, similarly to NGF, protected PC12 cells against serum deprivation-induced cell death, while TRKA inhibition with a selective TRKA inhibitor, GW174456, partially reversed the pro-survival effect of ENT-A010 (Figure 3).

**Figure 4:**
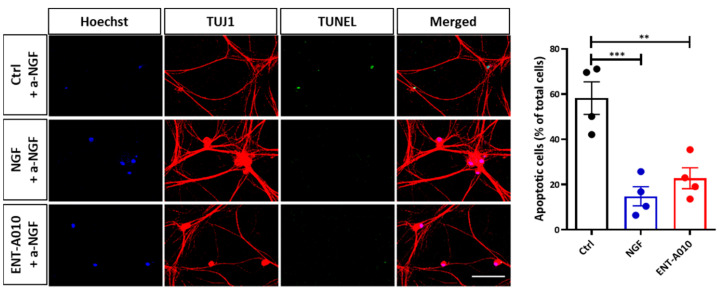
ENT-A010 promoted DRG neuron survival. DRG neurons were cultured for 2 days in a NGF-free and anti-NGF supplemented medium in the presence of NGF, ENT-A010 or control vehicle. Left panel: Cells stained for TUJ1 (red), with TUNEL (green) or Hoechst (blue). Scale bar: 100 μm (applies to all photos). Right panel: The quantification demonstrates the number of TUNEL^+^ (apoptotic) cells as % of Hoechst^+^ (total) cells. Data are shown as mean ± SEM, *n* = 4, **: *p* < 0.01, ***: *p* < 0.001.

Next, we set out to examine whether ENT-A010 affects the survival of DRG neurons, a neuronal population the survival of which is known to be highly dependent on NGF [53]. Primary DRG neurons were isolated from P0-P1 mouse pups and cultured for 14 days in the presence of NGF. Then, an NGF-free and anti-NGF supplemented medium was added to the cells for 2 days, thereby promoting DRG neuron cell death (Figure 4). ENT-A010, similarly to NGF treatment, prevented cell death of DRG neurons (Figure 4).

**Figure 5:**
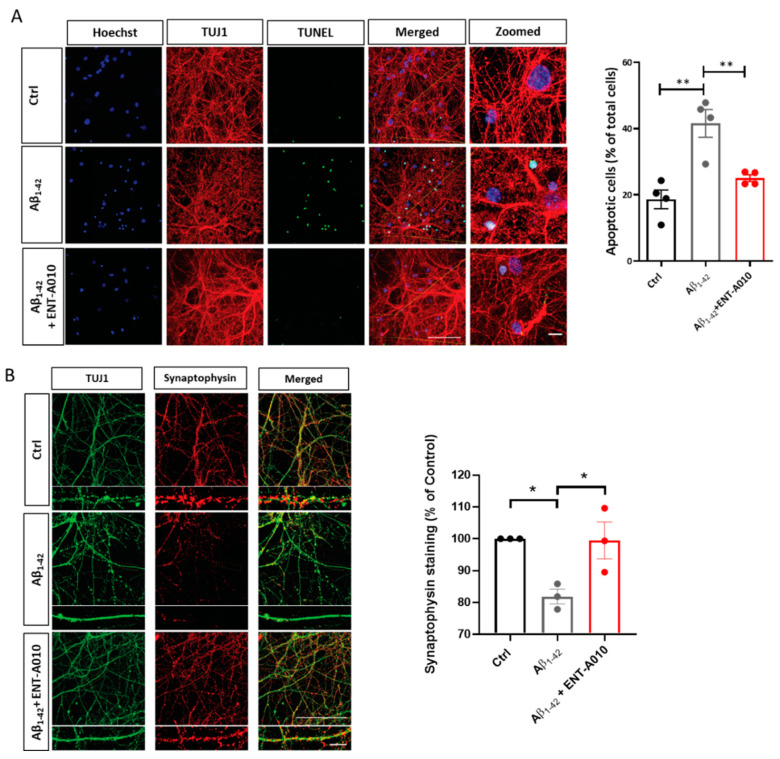
ENT-A010 protected primary hippocampal neurons against oligomeric Aβ toxicity. (**A**) Primary hippocampal neurons were treated for 48 h with 5 µM oligomeric Aβ and ENT-A010 (500 nM, freshly added every 24 h) or control vehicle and stained for TUJ1 (red), with TUNEL (green) or Hoechst (blue). The quantification shows the number of TUNEL^+^ (apoptotic) cells as % of the total number of cells. (**B**) Photomicrographs of primary hippocampal neurons, treated for 4 h with 5 µM oligomeric Aβ and ENT-A010 (500 nM) and immunostained against TUJ1 and Synaptophysin. The Synaptophysin- and TUJ1-positive areas were quantified and the ratio (Synaptophysin^+^ area /TUJ1^+^ area) was calculated and normalized in each experiment to the control. Ten to twelve images were acquired per sample. Scale bar: 100 μm (applies to all non-zoomed photos), and 10 μm (applies to all zoomed-in photos). Data are shown as mean ± SEM, *n* = 3–4, *: *p* < 0.05, **: *p* < 0.01.

Given that hippocampal neurons are prone to Aβ-induced cell death [55], we asked whether ENT-A010 can also preserve survival in toxic Aβ-challenged hippocampal neurons. We treated the latter with Aβ_1–42_ oligomers and ENT-A010 or control vehicles and found that ENT-A010 exerted strong protection against Aβ-induced cell death (Figure 5A). Moreover, we examined whether ENT-A010 can reverse Aβ-induced synapse degeneration [56]. To this end, hippocampal neurons were kept in culture for 16 days after isolation and treated for 4 h with Aβ_1–42_ oligomers and ENT-A010, followed by immunostaining for Synaptophysin, a pre-synaptic marker. ENT-A010 restrained Aβ-induced loss of Synaptophysin (Figure 5B).

### 3.3. ENT-A010 Promotes Phagocytosis in Microglia 

**Figure 6:**
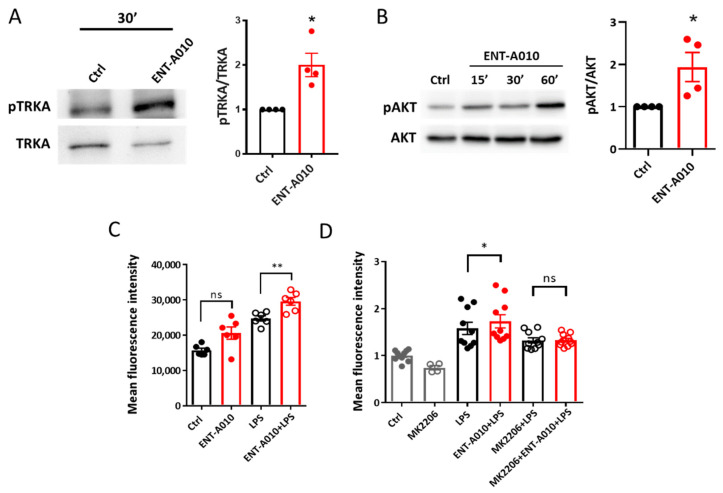
ENT-A010 induced TRKA and AKT signaling and promoted phagocytosis in microglial cells. (**A**) Primary microglia cells were treated for 30 min with 1 μM ENT-A010 or DMSO (Ctrl). Cell lysates were analyzed by WB for phospho- and total TRKA (**A**). (**B**) BV2 cells were treated for 15, 30 or 60 min with 1 μM ENT-A010 or for 60 min with DMSO (Ctrl) and analyzed for phospho- and total AKT by WB. A representative out of 4 experiments is shown (**A**,**B**). Signal intensities of pTRKA, total TRKA, pAKT and total AKT were measured and in each experiment, the ratio of pTRKA/TRKA and pAKT/AKT was set as 1 for Ctrl samples. (**B**) shows the quantification of pAKT/AKT at 60 min of treatment. (**C**,**D**) Primary microglia were treated with 1 μM ENT-A010 on two consecutive days, and 1 h after the second treatment, they were stimulated with 100 ng/mL LPS. In (**D**), cells were pre-treated with the AKT inhibitor MK2206 (2.5 µM) 30 min prior to the first ENT-A010 treatment. Twenty-four h after the LPS treatment, fluorescently labeled Aβ was applied for 2 h to the cells, followed by flow cytometry. Data are shown as mean ± SEM, *n* = 4 for (**A**), *n* = 4 for (**B**), *n* = 6 (**C**), *n* = 4–10 (**D**), *: *p* < 0.05, **: *p* < 0.01, ns: non-significant.

In vivo, the effects of drugs on neurons may be altered by their effects on microglia. Hence, we set out to examine whether ENT-A010, in addition to its neuroprotective effects, may also affect microglial functions, given that steroid hormones derive from and target microglia [11]. We previously showed that functional TRKA is expressed in microglia [38,43]. Here, we show that ENT-A010 induced TRKA and AKT phosphorylation in microglial cells (Figure 6A,B, respectively). NGF was previously shown to promote macropinocytosis and Aβ clearance in microglia [42]. Therefore, we assessed the effects of ENT-A010 on Aβ engulfment and found that it increased Aβ uptake in LPS-treated microglia (Figure 6C), while its effect was abolished by the AKT inhibitor MK2206 (Figure 6D). 

### 3.4. ENT-A010 Promotes a Protective Microglial Phenotype

**Figure 7:**
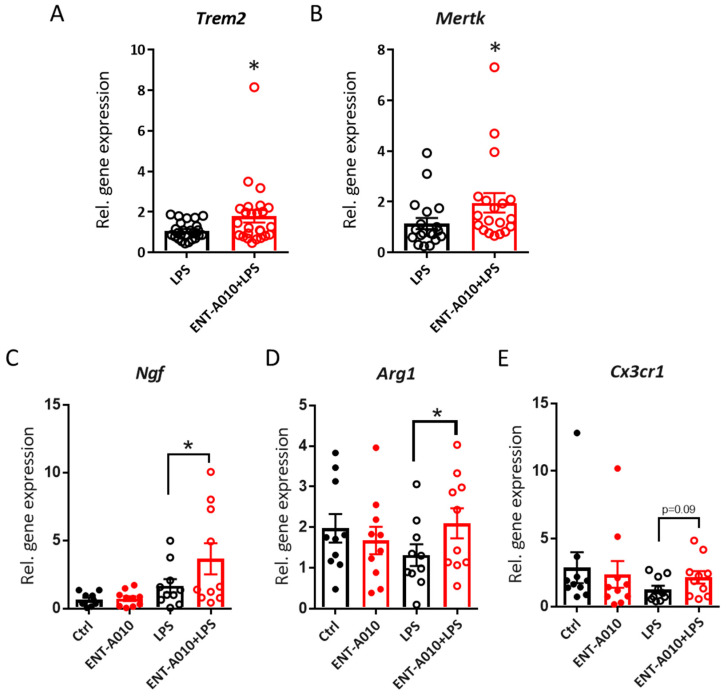
ENT-A010 promotes a protective microglial phenotype in vitro. Primary microglia were treated with 1 μM ENT-A010 on two consecutive days, and 1 h after the second treatment, they were stimulated with 100 ng/mL LPS. The expression of indicated genes was analyzed by qPCR. Data are shown as mean± SEM *n* = 25 for (**A**), *n* = 19 for (**B**), *n* = 10 for (**C**–**E**) *: *p* < 0.05.

Next, we examined whether ENT-A010 modulates the expression of *Triggering Receptor Expressed On Myeloid Cells 2* (*Trem2*) and *MER Proto-Oncogene, Tyrosine Kinase* (*Mertk*), which play a key role in phagocytosis [57,58,59,60]. ENT-A010 increased *Trem2* and *Mertk* expression in LPS-treated primary microglia (Figure 7A,B), standing in accordance with its effect on phagocytosis (Figure 6C,D). Moreover, it increased the expression of *Ngf* (Figure 7C), suggesting that ENT-A010-treated microglia may display enhanced neuroprotective function. In addition, ENT-A010 treatment enhanced the expression of *Arginase 1* (*Arg1*) in LPS-treated microglia, indicating that it may modulate arginine metabolism and promote an M2-like microglial phenotype (Figure 7D) [38]. Finally, ENT-A010-treated microglia showed a tendency for increased *Cx3cr1* expression, suggesting that ENT-A010 might restore the expression of homeostatic genes in inflammatory microglia (Figure 7E). We previously showed that TRKA activation by NGF or DHEA restrains inflammatory activation of LPS-stimulated microglia, as manifested by reduced secretion of TNF, IL-6 and IL-1β and decreased inducible nitric oxide synthase (iNOS) expression [38,43]. In contrast, ENT-A010 did not alter the LPS-induced expression of pro-inflammatory genes, such as *Il-1β*, *Tnf*, *Il-6,* or *iNos* (data not shown).

### 3.5. Peripherally Administered ENT-A010 Is Detected in the Brain

**Figure 8:**
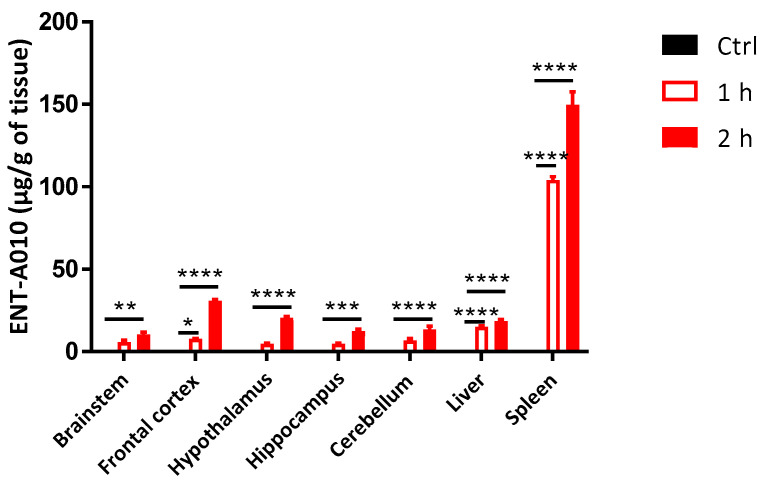
Peripherally administered ENT-A010 is detected in the brain. Mice were i.p. injected with 70 mg/kg ENT-A010 and 1 and 2 h later indicated brain regions, the liver and the spleen were collected, snap-frozen and analyzed by UHPLC-MS. Control mice were injected with the same amount of carrier solution (4.5% ethanol, 1% BSA, PBS). Data are shown as mean ± SEM, *n* = 3 mice per condition, *: adj *p* < 0.05 **: adj *p* < 0.01 ***: adj *p* < 0.005 ****: adj *p* < 0.0001.

Next, we set out to investigate the in vivo effects of ENT-A010. To assess whether peripherally administered ENT-A010 can reach the brain, mice were injected i.p. with 70 mg/kg ENT-A010 or control solution, and 1 or 2 h later, different brain regions (brainstem, frontal cortex, hypothalamus, hippocampus, cerebellum), livers and spleens were isolated and analyzed with UHPLC-MS. ENT-A010 was detected at both time points in all examined brain regions, as well as the liver and the spleen (Figure 8). 

### 3.6. ENT-A010 Preserves the Homeostatic Phenotype of Microglia in the Hippocampus

**Figure 9:**
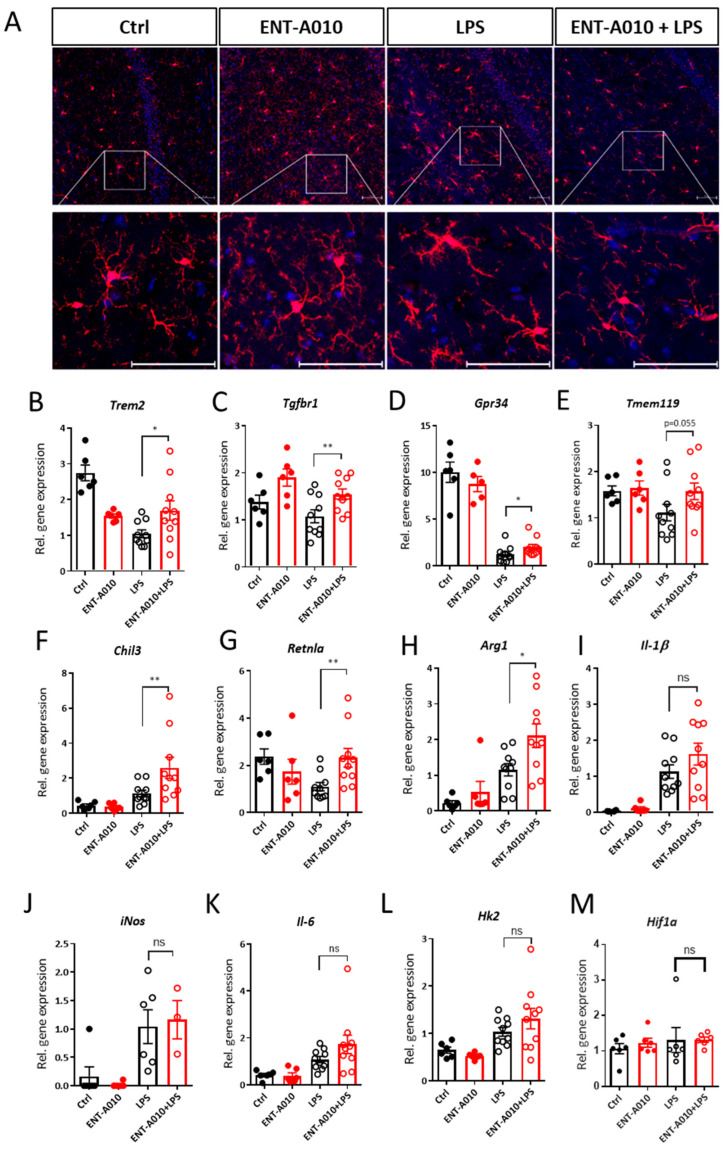
ENT-A010 restores microglial homeostatic features in the hippocampus of LPS-treated mice. (**A**) Mice were i.p. injected with 70 mg/kg ENT-A010 or control solution on two consecutive days. One h after the second treatment, they received i.p. 3 mg/kg LPS. After 16 h whole brains were isolated and immunostained against IBA1 (red) and with DAPI (blue). The whole hippocampal formation was imaged. Representative photomicrographs partially include the CA1 region, stratum radiatum and pyramidal layer (upper panel)—scale bars: 50 µm. (**B**–**M**). Hippocampi were isolated from mice treated as described in (**A**) and the whole RNA was analyzed by real-time PCR for indicated genes, using *18S* as a housekeeping gene. Fold change of relative gene expression was calculated based on the gene expression in the ‘LPS’ samples. Data are shown as mean ± SEM, *n* = 3–10 mice, *: *p* < 0.05, **: *p* < 0.01, ns: non-significant.

Next, we asked whether peripherally administered ENT-A010 may exert effects on the brain. To this end, mice received ENT-A010 i.p. on two consecutive days and 1 h after the second dose, they were treated i.p. with LPS for 16 h. We focused on the effects of ENT-A010 in the hippocampus, since the hippocampus is strongly affected by peripheral inflammation and prone to neurodegeneration [61,62,63], and asked whether ENT-A010 affected the phenotype of hippocampal microglia. Staining of brain sections (including the hippocampal formation) from mice treated with ENT-A010, LPS or ENT-A010 + LPS for the microglial marker ionized calcium-binding adaptor molecule 1 (IBA-1) demonstrated that LPS treatment induced thickening of the branches and enlargement of the cell body of microglia, standing in accordance with previous studies [64,65]. These microglial traits were blunted in the mice, which were pretreated with ENT-A010 (Figure 9A). Moreover, LPS decreased homeostatic gene expression signature, exemplified by reduced expression of *Trem2*, *Transforming growth factor beta receptor 1* (*Tgfbr1*), *G Protein-Coupled Receptor 34* (*Gpr34*) and *Transmembrane Protein 119* (*Tmem119*) (Figure 7B–E) [3,66]. Essentially, ENT-A010 partially restored the expression of these genes, suggesting that it may preserve the homeostatic signature of microglia in vivo (Figure 9B–E). In addition, it increased the expression of genes associated with the M2-like microglial phenotype, such as *Chitinase-like protein 3* (*Chil3*), *Resistin-like alpha* (*Retnla*) and *Arginase 1* (*Arg1*) (Figure 9F–H). In contrast, ENT-A010 treatment did not alter the expression of pro-inflammatory genes, such as *Il-1β*, *iNos*, *Il-6*, *Hexokinase 2* (*Hk2*) or *Hypoxia-inducible factor 1α* (*Hif1α*) (Figure 9I–M). Similarly, ENT-A010 did not affect *Il-1β*, *Il-6*, *Tnf* and *iNos* expression in livers and spleens in LPS-treated mice (Supplementary Figure S2). 

## 4. Discussion

Despite the significant advancement in deciphering the pathophysiology of neurodegenerative diseases, their treatment remains extremely challenging [67,68,69]. Neurotrophins, such as NGF, can prevent or reverse neurodegeneration, increase neurite outgrowth and promote synaptic plasticity [70,71]. Moreover, we and others have shown that NGF can exert anti-inflammatory effects and promote Aβ clearance in microglia [42,43]. Therefore, activation of NGF signaling could have the potential for the treatment of neurodegenerative diseases. However, being a large-sized protein, NGF has low stability in the circulation, negligible blood–brain barrier penetration and little diffusion within the CNS parenchyma [70,71,72,73,74], while it may cause hyperalgesia [75,76], thus limiting its pharmacological use. 

Small molecules, such as oligopeptides functioning as ligands of neurotrophin receptors, can modulate neurotrophin signaling and are able to ameliorate pathological features in neurodegenerative disease models [70,77,78,79]. Along the same line, we have demonstrated that steroid molecules can share similar functions with neurotrophins in terms of neuroprotection and anti-inflammatory effects and could be, therefore, interesting candidates for the treatment of CNS disorders involving neurodegeneration and neuroinflammation [11,25,26,27,28,29,30,38,39,40,41,80,81,82,83]. Specifically, the neurosteroid DHEA was shown to bind to TRKA receptor and activate downstream signaling in neuronal cells and microglia, thereby promoting neuronal survival and dampening microglia-mediated neuroinflammation [26,28,38]. Aiming to generate DHEA analogs that cannot be metabolized to androgen, a series of compounds were synthesized [39], out of which BNN-27 was proven to blunt neuronal apoptosis and promote survival through binding to the NGF receptors p75NTR and TRKA [40,41], downregulate microglial inflammatory responses [41,82] and increase oligodendrocyte survival in the cuprizone mouse model of demyelination [82]. 

Along the same line, we now synthesized ENT-A010 by replacing the metabolically labile oxirane ring of BNN-27 with the more stable cyclopropane moiety. ENT-A010 was selected from a panel of newly synthesized DHEA analogs (manuscript in preparation), based on its potential to induce TRKA phosphorylation. We show that ENT-A010 increased cell survival at comparable levels to NGF in serum-starved PC12 cells and NGF-starved DRG neurons. It also reduced apoptosis and restored Synaptophysin expression in Aβ-treated hippocampal neurons. Moreover, it increased Aβ uptake, favored microglial homeostatic signature, and increased *Ngf* expression in primary microglia. Both its neuroprotective effect and its effect on Aβ engulfment in microglia were blunted by TRKA inhibition.

Next, we examined the potential of peripherally given ENT-A010 to target brain tissues. ENT-A010 administered intraperitoneally was detected one and two hours post-injection in all tested brain regions (brainstem, frontal cortex, hypothalamus, hippocampus, cerebellum). Its brain levels were similar to those in the liver, signifying its efficient uptake in the brain compared to peripheral organs. Moreover, systemically administered ENT-A010 affected the morphology and transcriptional profile of hippocampal microglia in LPS-treated mice. In the experiments described here, we employed a previously used experimental setting, in which DHEA given prior to LPS restrained microglia-mediated neuroinflammation [38].

In this LPS model, we did not observe any neuronal apoptosis in the hippocampus evidenced by the absence of cleaved caspase 3 staining and no changes in the expression of apoptosis-regulating genes, such as *Bcl2* family genes, *Casp3* and *Apaf1* (data not shown); hence we did not study the neuroprotective effects of ENT-A010 in this model. AD, PD, or other neurodegenerative disease models should be used to delineate the neuroprotective role of ENT-A010 in vivo. Moreover, while pre-treatment relates to prevention, studies involving ENT-A010 post-treatment following an insult or disease onset will be important to examine the clinical relevance of these findings. 

Single-cell RNA analyses recently revealed a subset of microglia called disease-associated microglia (DAM) in AD, amyotrophic lateral sclerosis, multiple sclerosis and aging or models thereof [84,85,86]. DAM is featured by downregulated expression of homeostatic genes, such as *P2ry12*, *Tmem119* and *Cx3cr1*, and enhanced expression of phagocytic genes, including *Trem2* [3,84,85,87]. The current notion is that the clearing function of microglia plays a protective role in AD [86]. TREM2 mediates Aβ engulfment by microglia [57,58], is required for DAM activation [84] and limits AD progression [87], while a *TREM2* mutation is associated with increased AD risk [88].

The present study demonstrates that LPS-induced acute inflammation decreased the expression of homeostatic genes and *Trem2* in the hippocampus, standing in agreement with previous reports [89], while these effects were partially reversed by pretreatment with systemically applied ENT-A010. In accordance, ENT-A010 increased phagocytosis and Aβ clearance and enhanced *Trem2* and *Mertk* expression in microglia in vitro. These findings suggest that ENT-A010 may promote protective features in microglia. In contrast to NGF or DHEA, which also induce TRKA phosphorylation and exert anti-inflammatory effects in microglia [37,38,42,43], ENT-A010 did not affect *Il-1β*, *Il-6*, *Tnf*, *iNos,* or *Hk2* expression in LPS-treated microglia, which could be attributed to potential activation of different TRKA-dependent or independent signaling pathways by ENT-A010.

In conclusion, we developed a C17-spiro-cyclopropyl-DHEA synthetic analogue capable of inducing TRKA phosphorylation and AKT signaling, reaching all tested brain regions when applied peripherally, promoting neuronal survival, enhancing microglial phagocytic capacity and shifting hippocampal microglia towards a homeostatic state under inflammatory conditions. These traits could render ENT-A010 an interesting candidate for the treatment of neurodegenerative conditions, in which neuronal survival and microglial clearance capacity are compromised and microglial homeostasis is disrupted. To this end, a detailed exploration of the effects of ENT-A010 in animal models mimicking neurodegenerative diseases is essential. These research outcomes will shed light on the role of neuroactive steroid and neurotrophin mimetics on neurodegenerative and inflammatory aspects of CNS diseases. 

## Data Availability

The data supporting the findings of this study are available upon request.

## Supplementary Materials

The following are available online at https://www.mdpi.com/article/10.3390/biom12030424/s1, Table S1: Primer sequences, Figure S1: HPLC chromatogram of compound ENT-A010, Figure S2: ENT-A010 does not affect inflammatory gene expression in the liver and spleen of LPS-treated mice.
