# Supplementary material for: ENT-A010, a Novel Steroid Derivative, Displays Neuroprotective Functions and Modulates Microglial Responses
Source: Biomolecules. 2022 Mar 9;12(3):424. doi: 10.3390/biom12030424 (PMC8946810; doi:10.3390/biom12030424)
Supplement: Supplementary file 1 [file biomolecules-12-00424-s001.zip › biomolecules-1548210-supplementary.pdf]

Supplementary figures

Area % Report

Data File: C:\ChromQuest\Enterprise\Projects\Default\Method\TC\Alessia\ENT-A010_CH3CN_H2O_2.dat

Method: C:\ChromQuest\Enterprise\Projects\Default\Method\TC\Alessia\Gradient_CH3CN_H2O_2.met
Acquired: 10/4/2021 3:54:32 PM
Printed: 10/4/2021 4:22:23 PM

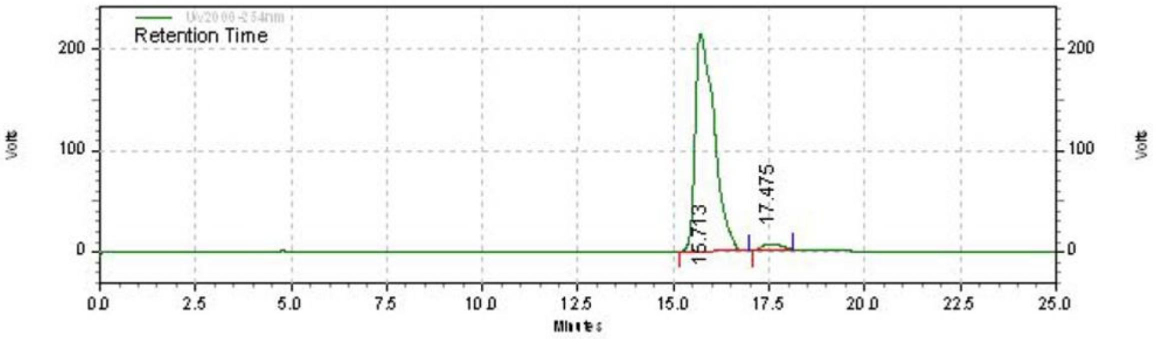

UV2000-254nm Results (System (10/4/2021 4:22:13 PM) (Reprocessed))

Retention Time	Area	Area %	Height	Height %
15.713	7461524	97.25	214472	97.19
17.475	211154	2.75	6196	2.81
Totals	7672678	100.00	220668	100.00

Figure S1. HPLC chromatogram of compound ENT-A010. The purity of ENT-A010 was determined by high-performance liquid chromatography (HPLC) using Nucelosil 100-5 C18 HD column, 5µm (4.6 x 250 mm), flow rate 1 mL/min, eluting with H2O-CH3CN gradient employing UV detection at 254 nm.

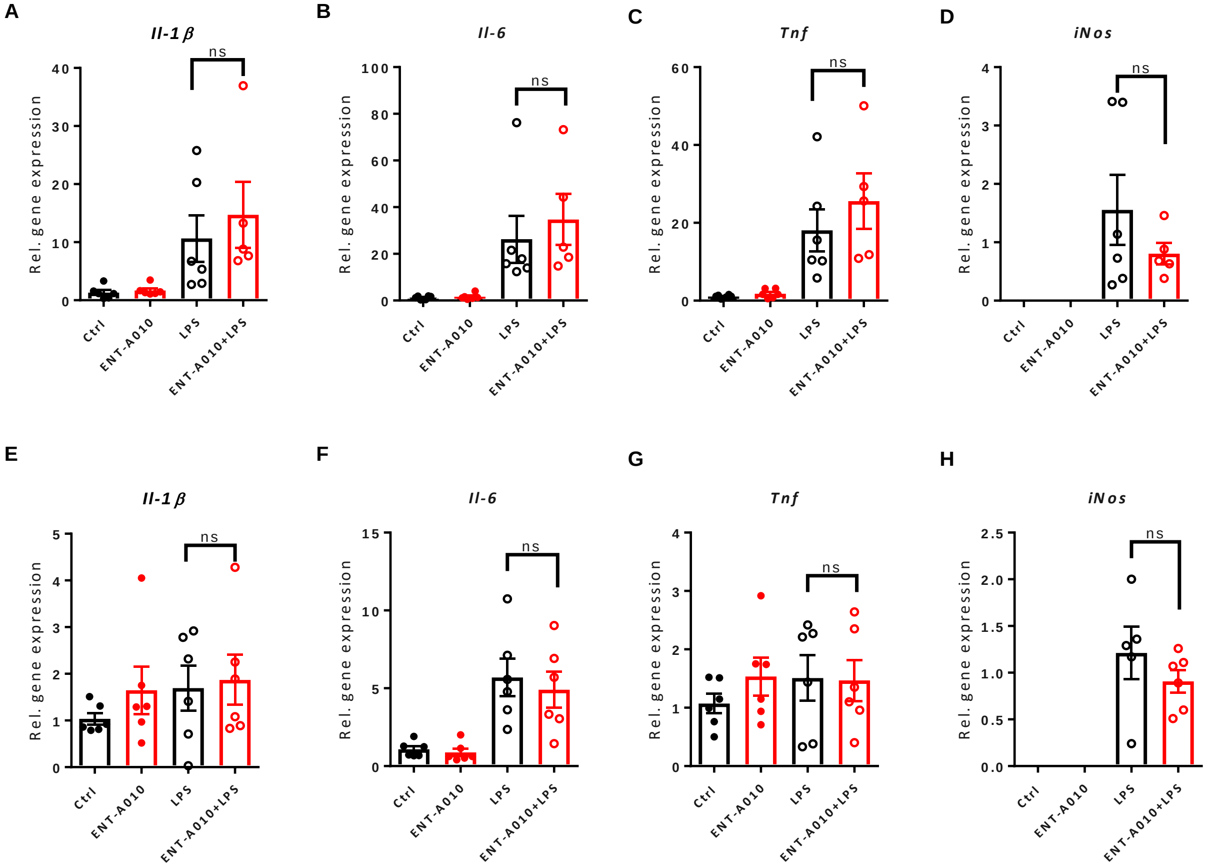

Figure S2. ENT-A010 does not affect inflammatory gene expression in the liver and spleen of LPS-treated mice. Mice were i.p. injected with 70 mg/kg ENT-A010 or control solution on two consecutive days. One h after the second treatment they received i.p. 3 mg/kg LPS. After 16 h livers (A-D) and spleens (E-G) were collected and whole RNA was analyzed by real-time PCR for indicated genes, using *18S* as a housekeeping gene. Gene expression in the 'Ctrl' group was set as 1. Data are shown as mean ± SEM, n=5-6 mice, ns: non-significant.

Table S1

Gene name	Forward primer sequence	Reverse primer sequence
18s	5′-GTTCCGACCATAAACGATGCC-3′	5′-TGGTGGTGCCCTTCCGTCAAT-3′
Trem2	5′-GTACTGGTGGAGGTGCTGGA-3′	5′-GGAGGTGCTGTGTTCCACTT-3′
Mertk	5′-CGGTAATAATCACCACTGTAAATCTTTCT-3′	5′-TTGCGGGATGACATGACTGT-3′
Tgfbr1	5′-TGCTCCAAACCACAGAGTAGGC-3′	5′-CCCAGAACACTAAGCCCATTGC-3′
Gpr34	5′-GTGCCAAATGTCACTAGTAGCTGTCC-3′	5′-TCCAACCAGTCCCACGAGGAAT-3′
Tmem119	5′-ACCCAGAGCTGGTTCCATAG-3′	5′-GCCGGGAGTGACACAGAGTA-3′
Chil3	5′-TTCTGAATGAAGGAGCCACTGA-3′	5′-ACCACGGCACCTCCTCCTAAATTG-3′
Retnla	5′-CTGCTACTGGGTGTGCTTGTG-3′	5′-GCAGTGGTCCAGTCAACGAG-3′
Arg1	5′-GAACACGGCGGCAGTGGCTTTAAC-3′	5′-TGCTTAGCTCTGTCTGCTTTGC-3′
Il-1β	5′-TGGGATGATGATGATAACCTGC-3′	5′-TCGTTGCTTGGTTCTCCTTGTA-3′
iNos	5′-TGCAGCAGAAGTGCAAAGTCTC-3′	5′-GCCATTTTGGTGACTCTTAGGG-3′
Il-6	5′-CCTTCCTACCCCAATTTCCAAT-3′	5′-AACGCACTAGGTTTGCCGAGTA-3′
Hk2	5′-ATCGCCTGCTTATTCACGGA-3′	5′-TGAGAGACGCATGTGGTAGAG-3′
Hif1a	5′-CTGTTAGGCTGGGAAAAAGTTA-3′	5′-ACCTTCATCGGAAACTCCAAAG-3′
Ngf	5′-CATGGGGGAGTTCTCAGTGT-3′	5′-GCACCCACTCTCAACAGGAT-3′
Cx3cr1	5′-AGGACACAGCCAGACAAG-3′	5′-TCAGGGGAGAAAGCAAG-3′
